# Phylogenetic evidence of possible zoonotic circulation of *Leptospira* species between human febrile patients and bats within a same interface

**DOI:** 10.1016/j.onehlt.2026.101382

**Published:** 2026-03-03

**Authors:** J. Manuel Matiz-González, Carlos Ramiro Silva-Ramos, Piedad Agudelo-Flórez, Elsio A. Wunder, Marylin Hidalgo

**Affiliations:** a Molecular Genetics and Antimicrobial Resistance Unit, Universidad El Bosque, Bogotá, Colombia; b Grupo de Enfermedades Infecciosas, Departamento de Microbiología, Facultad de Ciencias, Pontificia Universidad Javeriana, Bogotá, Colombia; c Department of Pathology, University of Texas Medical Branch, Galveston, TX, United States; d Programa de Enfermería, Facultad de Ciencias de la Salud, Universidad Distrital Francisco José de Caldas, Bogotá, Colombia; e Grupo de Investigación en Ciencias de la Vida y la Salud, Escuela de Graduados, Universidad CES, Medellín, Colombia; f Department of Pathobiology and Veterinary Science, University of Connecticut, Storrs, CT, USA; g Gonçalo Moniz Institute, Oswaldo Cruz Foundation, Brazilian Ministry of Health, Salvador, Brazil

**Keywords:** Leptospirosis, Chiroptera, 16S rRNA gene, Molecular phylogenetics, Acute undifferentiated febrile illness, Wildlife, One health

## Abstract

Leptospirosis is a widespread zoonotic disease caused by pathogenic *Leptospira* spp., maintained by a wide range of animal reservoirs. In Colombia, it remains a major cause of acute undifferentiated febrile illness, yet the role of wildlife, particularly bats, in its transmission cycle is poorly understood. To investigate the relationship between *Leptospira* infecting humans and bats in Villeta, Colombia, thirty partial available leptospiral 16S rRNA sequences from febrile patients (*n* = 13) and bats (*n* = 17) were analyzed. Hierarchical clustering identified twelve groups (A–L), two (C and G) containing sequences from both hosts, showing 99–100% identity. Phylogenetic analysis placed these clusters within the P1 clade, forming distinct monophyletic groups separate from known species, suggesting *Leptospira* lineages shared between bats and febrile patients. These findings provide molecular evidence suggesting a genetic relationship between *Leptospira* species identified from febrile patients and bats within a shared ecological interface in Colombia.

## 1 Introduction

Pathogenic *Leptospira* species cause leptospirosis, a neglected zoonotic disease of global distribution and a major public health concern in tropical regions worldwide, particularly in developing and underdeveloped countries [1]. These long, thin, and highly motile spirochetes comprise 74 species, 43 of them potentially pathogenic to humans and several animal species (P1 and P2 clades) and 31 being free-living saprophytic species (S1 and S2 clades) [2], [3]. *Leptospira* is a frequent cause of acute undifferentiated febrile illness (AUFI), and in some regions surpasses common etiologies such as malaria and dengue [1].

Pathogenic *Leptospira* species are maintained among a wide range of domestic and wild animals, which develop persistent renal colonization and shed the bacteria in urine. Considering that *Leptospira* can survive long periods in the environment, contaminated water and soil become sources of spillover to humans and other susceptible animals [4]. Although rodents are traditionally considered the most important reservoirs, other wildlife species may also play significant role in the eco-epidemiology of leptospirosis, especially in rural settings [4], [5]. Increased human-driven activities such as uncontrolled agriculture, accelerated urbanization, and eco-tourism, along with climate change, have intensified human-wildlife interactions, favoring re-emergence and spread of zoonoses, including leptospirosis [6].

Among wildlife, bats are particularly affected by environmental disturbances due to their ecological plasticity, which allows them to adapt to urban environments and become synanthropic [7]. Their unique biological traits make them critical reservoirs and shedders of several microorganisms [7]. Although their role in leptospirosis is not yet fully understood, evidence demonstrates that they harbor a wide diversity of pathogenic *Leptospira* species [8], [9], with reports of human infections associated with bat exposure [10].

Humans can acquire leptospirosis either through direct contact with infected animals or, more commonly, by indirect contact with contaminated soil or water [11]. The highest risk remains among people living in resource-limited areas of developing and underdeveloped countries, particularly rural population engaged in livestock and agriculture, as well as individuals participating in eco-tourism in tropical regions [7], [11]. However, with the increase of social inequality worldwide, developed countries are also experiencing increased leptospirosis cases, especially among underprivileged populations [12].

Colombia provides an ideal setting to study these dynamics, as approximately 85% of its territory consists of tropical ecosystems, underscoring the importance of tropical diseases for both residents and visitors. In Villeta municipality, an AUFI-endemic region, *Leptospira* is a major causative agent [13], with *Leptospira santarosai* and two additional uncharacterized species identified as etiological agents [14]. Sources of infection in the area remain largely unexplored, with only one study in local wildlife reporting the detection of *Leptospira* spp. in bats closely related to pathogenic species [15]. However, there are no studies exploring potential links between species causing leptospirosis and those circulating in local wildlife. Therefore, the aim of the present study was to assess the genetic relationship between *Leptospira* sequences identified from febrile patients and bats within a same interface.

## 2 Methods

Villeta municipality, Cundinamarca department (5°00′46″ N, 74°28′23″ W) is located 84 km from Bogotá D.C. at 850 m above sea level. It covers 140 km^2^ and has an average temperature of 26 °C and relative humidity of 80–97%.

Partial *Leptospira* 16S rRNA gene sequences used for comparative molecular analyses in the present study were retrieved from the GenBank database. These sequences belonged to samples generated in two previous independent studies performed in the same region which involved febrile human patients [14] and bat specimens [15], from which *Leptospira* DNA had been molecularly detected.

Multiple sequence alignment was performed between *Leptospira* sequences obtained from febrile patients and bats using the ClustalW algorithm [16]. The identity matrix was used to cluster the sequences with the “hclust” and “cutree” functions (hierarchical clustering) from the Stats package in R, as recommended for clustering 16S rRNA sequences from the same species [17]. Sequences from mixed clusters containing both human and bat sequences were aligned with 16S rRNA sequences from reported *Leptospira* species (Supplementary Table S1) using ClustalW. Subsequently, a maximum likelihood (ML) phylogeny was constructed in MEGA 12 [18], using Kimura-2-parameter model with gamma distribution and invariant site analysis, and a branch support analysis with 1000 bootstrap iterations.

## 3 Results

**Fig. 1:**
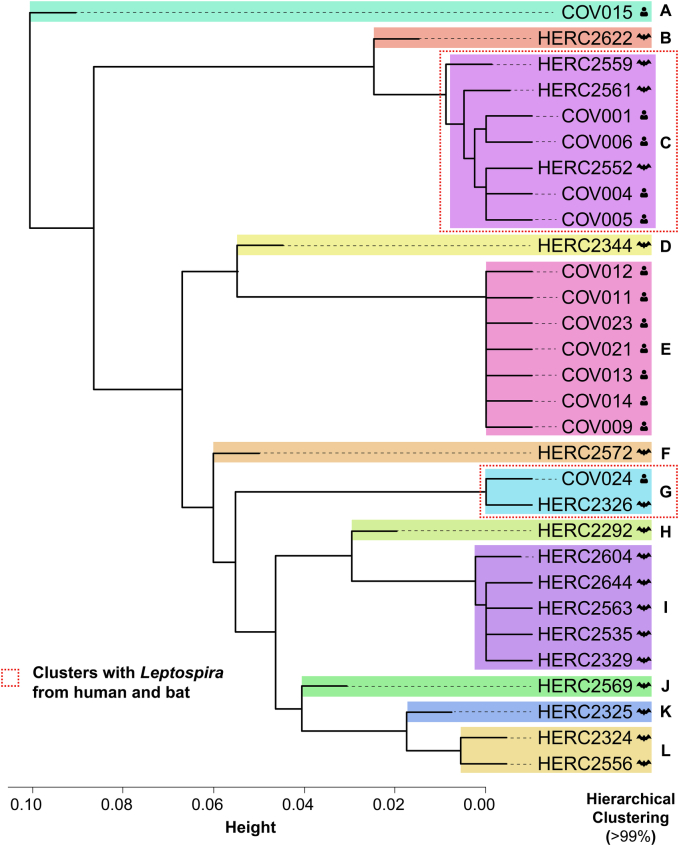
Hierarchical clustering among partial 16S rRNA sequences obtained from febrile human patients and bats from Villeta, Colombia. Each cluster is denoted by a colored box and an indicative letter. Sequences obtained from bats and humans are denoted by an icon indicating the host. A threshold of 99% was used for clustering.

Thirty *Leptospira* 16S rRNA sequences retrieved from GenBank, 13 originally obtained from febrile human patients and the remaining 17 from bats captured in the same municipality, were analyzed using hierarchical clustering. The main demographic and clinical characteristics of the human patients from whom the sequences were obtained are summarized in Supplementary Table S2. A total of twelve clusters (A–L) were identified, two of which (C and G) included sequences from both febrile human patients and bats (Fig. 1). Cluster C was composed of three bat-derived and four human-derived sequences, showing high identity values ranging between 99% to 100%; whereas Clade G comprised one sequence from each host, both exhibiting 100% identity (Table 1), indicating a close genetic relationship between *Leptospira* detected in febrile human patients and bats.

**Table 1:**
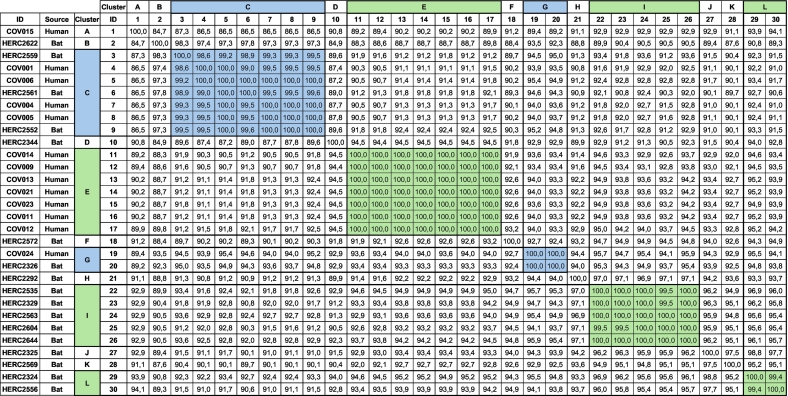
Percentage identity matrix among 16S rRNA sequencing from humans and bats. Blue clusters: clusters composed of more than one sequence from a single host. Green clusters: clusters composed of more than one sequence from both hosts.

**Fig. 2:**
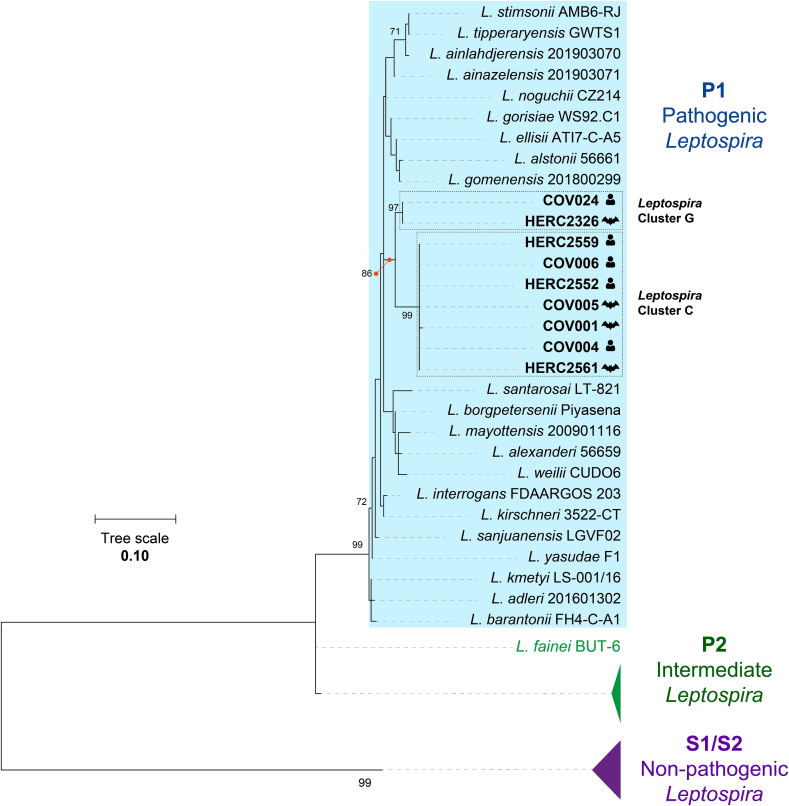
Phylogeny of 16S rRNA of the genus *Leptospira*, including *Leptospira* clusters composed by sequences from febrile human patients and bats (C and G). Sequences from humans and bats are signaled with icons indicating the respective host. *Leptospira* clades C and G are indicated by dashed boxes. P1 *Leptospira* spp. group is indicated by a blue colored box. P2 and S1/S2 *Leptospira* spp. groups were comprised and signaled with green and purple triangles respectively. Only bootstrap values greater than 70 are shown. (For interpretation of the references to colour in this figure legend, the reader is referred to the web version of this article.)

To unveil the genetic relationship between these two *Leptospira* clusters and previously reported species [14], [15], we reconstructed a *Leptospira* 16S rRNA phylogeny using reference genome sequence of all species available in NCBI. Both clusters grouped within the pathogenic *Leptospira* clade P1, each forming highly supported bootstrap clades with shorter branch distance than those observed among distinct species (Fig. 2). These results indicate a close genetic relationship between human- and bat-derived sequences in both groups, supporting their classification as the same species. Moreover, both clusters were positioned on a separate branch unrelated to any *Leptospira* species (Fig. 2), suggesting them as novel species associated with bat and human hosts.

## 4 Discussion

In Colombia, Villeta has been recognized as a hotspot for several AUFI etiologies, with leptospirosis representing a significant public health burden [13]. Leptospirosis is a notifiable disease in Colombia since 2007, and cases continue to fluctuate, indicating a major and current public health concern. This persistent burden is largely due to the complex transmission cycle of pathogenic *Leptospira* species involving the environment and multiple animal reservoirs [4]; therefore, identifying natural infection sources is essential for designing interventions to reduce the number of cases. By comparing partial *Leptospira* sequences from febrile patients and bats, we found a high degree of similarity among some sequences, suggesting shared genetic *Leptospira* lineages between both species from the same region. However, given that only a subset of sequences clustered together, it is not possible to infer that bats are the source, or only source, of human infections. These findings also raise the possibility that both humans and bats are being infected separately through exposure to a common environmental source of *Leptospira*, a hypothesis that warrants further investigation.

Although previous studies have identified human-pathogenic *Leptospira* in Colombian bats [15], to our knowledge, this study provides the first molecular evidence of genetic relationship between sequences obtained from bats and febrile patients within the same geographic region. Bats harbor a substantial diversity of pathogenic *Leptospira* (P1), including species related to human leptospirosis [8]. Additionally, a case report has implicated bats in human leptospirosis by describing a serologically confirmed infection after direct contact with a bat in a swimming pool [10]. Moreover, available evidence documented chronic renal colonization and urinary shedding in bats [19]. Together, all these findings strongly support the significant eco-epidemiological role that bats may play in leptospirosis transmission cycle.

Data from the present study suggest that in endemic tropical regions, such as Villeta, exposure to bats may represent a risk factor for *Leptospira* infection. Human-bat interactions has been documented in nearby municipalities (∼50 km) such as San Sebastián de Mariquita, Armero Guayabal, and Ambalema, reporting bats entering homes, workplaces, and roosting in trees close to human activity [20]. Therefore, clinicians should inquire about bat exposure during AUFI patients' anamnesis. Since contact with environmental sources contaminated by bat urine (e.g., fruits, soil, or surface water) may be more frequent, public health actions should aim to protect bat habitats while minimizing the risk of human infection.

Notably, direct contact with bats was not explicitly reported by any of the patients from whom these sequences were obtained [13], [14]. Nevertheless, the clustering of human- and bat-derived sequences supports the co-circulation of related *Leptospira* lineages in wildlife and human-associated environments. Although the directionality and mechanisms of transmission cannot be determined from these data, these findings highlight the complexity of *Leptospira* ecology and underscores the need for integrated studies to disentangle the roles of wildlife, environmental sources, and intermediate hosts. The fact that human sequences genetically related to those from bats were associated with clinically significant disease, including a confirmed fatal case (Supplementary Table S2), underscores the public health relevance of these *Leptospira* species circulating in bat populations.

Although febrile patients and bats derived sequences showed high similarity, sometimes identical, our analysis relied on a partial region of the 16S rRNA fragment. While this marker is widely used for assessing *Leptospira* species diversity, it has limited discriminatory power for strain-level identification [21]. Further studies involving *Leptospira* isolation, full-genome sequencing, or metagenomics are needed to confirm these associations.

In this context, the detection of highly similar *Leptospira* sequences in humans and bats within the same ecological interface reinforces the importance of adopting a One Health approach to leptospirosis surveillance in endemic regions. Integrated strategies combining wildlife monitoring, environmental sampling, and clinical surveillance could improve early detection of circulating pathogenic lineages and inform targeted prevention measures. Strengthening collaboration between public health authorities, ecologists, and local communities may also contribute to risk communication strategies that reduce human exposure while maintaining ecological balance. Importantly, bats should not be regarded solely as sources of infection, as they provide essential ecosystem services such as insect population control, pollination, and seed dispersal [22]. Public health interventions should therefore prioritize minimizing risky human–wildlife interactions while ensuring the conservation of bat populations and their habitats. Ultimately, understanding the interconnected roles of wildlife, environment, and human activity is essential for designing sustainable interventions aimed at reducing leptospirosis burden in tropical settings.

## 5 Conclusions

This study provides molecular evidence supporting a close genetic relationship between *Leptospira* spp. infecting humans and bats in Villeta, Colombia. The identification of highly similar, and in some cases identical, partial 16S rRNA gene sequences from both hosts suggests possible shared transmission pathways within the same ecological interface. Beyond documenting genetic similarity, these findings reinforce the relevance of bats within a local One Health framework and underscore the need to further investigate their role in the eco-epidemiology of leptospirosis. These findings support the need for future studies incorporating microbial isolation, whole-genome approaches, broader sampling strategies, and complementary epidemiological data which are essential to better understand and clarify transmission dynamics, assess reservoir competence, and evaluate the public health relevance of bat-associated *Leptospira* in endemic settings.

The following are the supplementary data related to this article.

Supplementary Table 1 Accession IDs and metadata for the sequences used in the study Supplementary Table 1

Supplementary Table 2 Summary of demographic characteristics, epidemiological information, and clinical features of febrile patients whose *Leptospira* 16S rRNA sequences clustered with bat-derived sequences (clusters C and G). Supplementary Table 2

## CRediT authorship contribution statement

**J. Manuel Matiz-González:** Writing – original draft, Visualization, Validation, Methodology, Investigation, Formal analysis, Data curation, Conceptualization. **Carlos Ramiro Silva-Ramos:** Writing – review & editing, Writing – original draft, Validation, Supervision, Project administration, Methodology, Formal analysis, Conceptualization. **Piedad Agudelo-Flórez:** Writing – review & editing, Validation, Formal analysis. **Elsio A. Wunder:** Writing – review & editing, Validation, Formal analysis. **Marylin Hidalgo:** Writing – review & editing, Resources, Project administration, Funding acquisition, Formal analysis.

## Funding

This work was funded by the Fogarty International Center of the National Institutes of Health under Award Number D43TW010331. The content is solely the responsibility of the authors and does not necessarily represent the official views of the National Institutes of Health.

## Declaration of competing interest

The authors declare that they have no known competing financial interests or personal relationships that could have appeared to influence the work reported in this paper.

## Data Availability

All sequence data used in this study are publicly available in the GenBank database, and accession numbers are provided in the manuscript.

## References

[1] Rajapakse S., Fernando N., Dreyfus A., Smith C., Rodrigo C. (2025). Leptospirosis. Nat. Rev. Dis. Primers.

[2] Hamond C., Tibbs-Cortes B., Fernandes L.G.V., LeCount K., Putz E.J., Anderson T., Camp P., Stuber T., Hicks J., van der Linden H., dos Santos Ribeiro P., Bayles D.O., Schlater L.K., Nally J.E. (2025). *Leptospira gorisiae* sp. nov., *L. cinconiae* sp. nov., *L. mgodei* sp. nov., *L. milleri* sp. nov. and *L. iowaensis* sp. nov.: five new species isolated from water sources in the Midwestern United States. Int. J. Syst. Evol. Microbiol..

[3] Vincent A.T., Schiettekatte O., Goarant C., Neela V.K., Bernet E., Thibeaux R., Ismail N., Mohd Khalid M.K.N., Amran F., Masuzawa T., Nakao R., Amara Korba A., Bourhy P., Veyrier F.J., Picardeau M. (2019). Revisiting the taxonomy and evolution of pathogenicity of the genus *Leptospira* through the prism of genomics. PLoS Negl. Trop. Dis..

[4] Cilia G., Bertelloni F., Fratini F. (2020). Leptospira infections in domestic and wild animals. Pathogens.

[5] Byrne A.W., Morgan E.R. (2024). Emerging and endemic infections in wildlife: epidemiology, ecology and management in a changing world. Pathogens.

[6] Esposito M.M., Turku S., Lehrfield L., Shoman A. (2023). The impact of human activities on zoonotic infection transmissions. Animals.

[7] Biswas R., Debnath C., Samanta I., Barua R., Singh A.D. (2020). Ecology of bats and their role in emerging zoonotic diseases: a review. Rev. Sci. Tech. (OIE).

[8] Matiz-González J.M., Ballesteros-Ballesteros J.A., Hernández M., Mejorano-Fonseca J.A., Cuervo C., Faccini-Martínez Á.A., Hidalgo M., Pérez-Torres J., Silva-Ramos C.R. (2024). Genetic diversity of P1/pathogenic Leptospira species hosted by bats worldwide. Zoonoses Public Health.

[9] Esteves S.B., Gaeta N.C., Batista J.M.N., Dias R.A., Heinemann M.B. (2022). *Leptospira* sp. infection in bats: a systematic review and meta-analysis. Transbound. Emerg. Dis..

[10] Vashi N.A., Reddy P., Wayne D.B., Sabin B. (2010). Bat-associated leptospirosis. J. Gen. Intern. Med..

[11] Mwachui M.A., Crump L., Hartskeerl R., Zinsstag J., Hattendorf J. (2015). Environmental and behavioural determinants of leptospirosis transmission: a systematic review. PLoS Negl. Trop. Dis..

[12] Atherstone C., Galloway R., Schafer I., Artus A., Rodriguez M.M., Ryff K., Rivera A.M., Slavinski S., Paladini M., Kemble S.K., Berreman J.M., Kallas G., Traxler R., Kharod G., Guerra M., Stoddard R.A., Moore H., Bower W.A., Negron M.E., DeBord K. (2025). Epidemiological, temporal, and geographic trends of leptospirosis in the United States, 2014–2020. PLoS Negl. Trop. Dis..

[13] Silva-Ramos C.R., Gil-Mora J., Serna-Rivera C.C., Martínez Díaz H.C., Restrepo-López N., Agudelo-Flórez P., Arboleda M., Díaz F.J., Faccini-Martínez Á.A., Hidalgo M., Melby P.C., Aguilar P.V., Cabada M.M., Tobón-Castaño A., Rodas J.D., members of the GIDRN – global infectious diseases research network (2023). Etiological characterization of acute undifferentiated febrile illness in Apartadó and Villeta municipalities, Colombia, during COVID-19 pandemic. Inf. Med..

[14] Silva-Ramos C.R., Matiz-González J.M., Gil-Mora J., Martínez Díaz H.C., Faccini-Martínez Á.A., Cuervo C., Melby P.C., Aguilar P.V., Cabada M.M., Rodas J.D., Hidalgo M. (2024). Molecular characterization of Leptospira species among patients with acute undifferentiated febrile illness from the municipality of Villeta, Colombia. Trop. Med. Infect. Dis..

[15] Silva-Ramos C.R., Matiz-González J.M., Barrero-Rubiano C.A., Villar J.D., Cuéllar-Sáenz J.A., López-Rivera C., Robayo-Sánchez L.N., Henao-Osorio J.J., Cardona-Giraldo A., Mejorano-Fonseca J.A., Agudelo-Flórez P., Cortés-Vecino J.A., Faccini-Martínez Á.A., Cuervo C., Ramírez-Chaves H.E., Hidalgo M., Ramírez-Hernández A. (2025). Molecular detection and characterization of Leptospira species in bats and other small wild mammals from Villeta municipality, Colombia. Comp. Immunol. Microbiol. Infect. Dis..

[16] Thompson J.D., Higgins D.G., Gibson T.J. (1994). CLUSTAL W: improving the sensitivity of progressive multiple sequence alignment through sequence weighting, position-specific gap penalties and weight matrix choice. Nucleic Acids Res..

[17] Rodriguez-R L.M., Castro J.C., Kyrpides N.C., Cole J.R., Tiedje J.M., Konstantinidis K.T. (2018). How much do rRNA gene surveys underestimate extant bacterial diversity?. Appl. Environ. Microbiol..

[18] Kumar S., Stecher G., Suleski M., Sanderford M., Sharma S., Tamura K. (2024). MEGA12: molecular evolutionary genetic analysis version 12 for adaptive and green computing. Mol. Biol. Evol..

[19] Seidlova V., Nemcova M., Pikula J., Bartonička T., Ghazaryan A., Heger T., Kokurewicz T., Orlov O.L., Patra S., Piacek V., Treml F., Zukalova K., Zukal J. (2021). Urinary shedding of leptospires in palearctic bats. Transbound. Emerg. Dis..

[20] Ramírez-Fráncel L.A., García-Herrera L.V., Guevara G., Losada-Prado S., Lim B.K., Villa-Navarro F.A., Reinoso-Flórez G. (2021). Human–bat interactions in Central Colombia: regional perceptions of a worldwide fragile life zone. Ethnobiol. Conserv..

[21] Johnson J.S., Spakowicz D.J., Hong B.Y., Petersen L.M., Demkowicz P., Chen L., Leopold S.R., Hanson B.M., Agresta H.O., Gerstein M., Sodergren E., Weinstock G.M. (2019). Evaluation of 16S rRNA gene sequencing for species- and strain-level microbiome analysis. Nat. Commun..

[22] Kasso M., Balakrishnan M. (2013). Ecological and economic importance of bats (order Chiroptera). Int. Sch. Res. Not..

