# Supplementary material for: Phylogenetic evidence of possible zoonotic circulation of Leptospira species between human febrile patients and bats within a same interface
Source: One Health. 2026 Mar 3;22:101382. doi: 10.1016/j.onehlt.2026.101382 (PMC12993018; doi:10.1016/j.onehlt.2026.101382)
Supplement: Supplementary Table 2 — Summary of demographic characteristics, epidemiological information, and clinical features of febrile patients whose Leptospira 16S rRNA sequences clustered with bat-derived sequences (clusters C and G). [file mmc2.docx]

**Table S2.** Summary of febrile patient demographics, epidemiological data and clinical features

| **Sample ID** | **Age group** | **Gender** | **Origin** | **Occupation** | **Recruitment month** | **Exposure history** | **Fever characteristics** | **Other clinical manifestations** | **Outcome** |
| --- | --- | --- | --- | --- | --- | --- | --- | --- | --- |
| COV001 | Young adult (25) | Male | Rural | Construction and general works | September | Mosquitoes, rodents, pigs | 6-day fever, sudden onset, intermittent, associated with total inactivity | Chills, malaise, anorexia, insomnia, sweats, fatigue, retro-ocular pain, cough, diarrhea, myalgia, arthralgia, headache | Discharged and resolved |
| COV004 | Middle-age adult (31) | Male | Urban | Disemployed | September | Mosquitoes, ticks, fleas, lice, rodents, cattle, horses, pigs, caprines, birds, dogs, cats | 2-day fever, gradual onset, intermittent, associated with normal activity | Chills, malaise, anorexia, insomnia, fatigue, oral ulcer, cough, nausea, abdominal pain, diarrhea, macular rash, myalgia | Transferred to a higher level hospital |
| COV005 | Old adult (55) | Male | Urban | Transport and deliveries | September | Mosquitoes, dogs | 4-day fever, gradual onset, intermittent, associated with partial inactivity | Chills, malaise, anorexia, insomnia, sweats, fatigue, cough, nausea, diarrhea, coluria, vomiting, headache, hepatomegaly | Fatal outcome |
| COV006 | Children (11) | Female | Rural | Student | September | Mosquitoes, ticks, fleas, lice, rodents, dogs, cats | 2-day fever, sudden onset, intermittent, associated with partial inactivity | Chills, malaise, insomnia, sweats, fatigue, cough, nausea, abdominal pain, coluria, vomiting, constipation, arthralgia, headache | Discharged and resolved |
| COV024 | Young adult (24) | Male | Urban | Transport and deliveries | December | Mosquitoes | 1-day fever, sudden onset, continuous, associated with normal activity | Chills, malaise, sweats, fatigue, nausea, | Discharged and resolved |
